# Treatment of Hemiplegia

**Published:** 1831-04-01

**Authors:** 


### II. Treatment of Hemiplegia.

Dr. Prichard, of Bristol, has drawn the attention of the profession to a mode of treating old and obstinate hemiplegia, and other diseases of the head, which he has found more successful than any other. He properly observes that recent cases are to be treated by antiphlogistics and local depletion, together with a cautious use of mercury; but, after these measures have been pursued for a certain time, depletory remedies can no longer be pursued—and counter irritation, with a drain from the vicinity of the disease, is most likely to be beneficial. For this purpose, Dr. P. makes an incision in the line of the sagittal suture, or posterior to that, and fills the incision with peas, which are to be renewed and kept discharging for a considerable time. He has employed this process with much advantage in many other affections of the head besides hemiplegia—more especially in cases of stupor, or coma occurring in the course of severe typhoid fevers. Many years ago, we were in the habit of using a somewhat similar remedy in cases of epilepsy. The head being well shaved, a narrow strip of lytta plaster was applied in the line of the sagittal suture, and when the skin was removed, a cord of silk or cotton thread, imbued with blistering ointment, was laid along the above line and daily renewed. This kept up a constant purulent discharge from the part, and is of extremely easy application.—*Med. Gaz.*

